# Persistence of Memory and the Comma Bacillus

**DOI:** 10.3201/eid1711.AC1711

**Published:** 2011-11

**Authors:** Polyxeni Potter

**Affiliations:** Author affiliation: Centers for Disease Control and Prevention, Atlanta, Georgia, USA

**Keywords:** art science connection, emerging infectious diseases, art and medicine, Georges Desarmes, The Bathers, Haiti, cholera, persistence of memory and the comma bacillus

**Figure:**
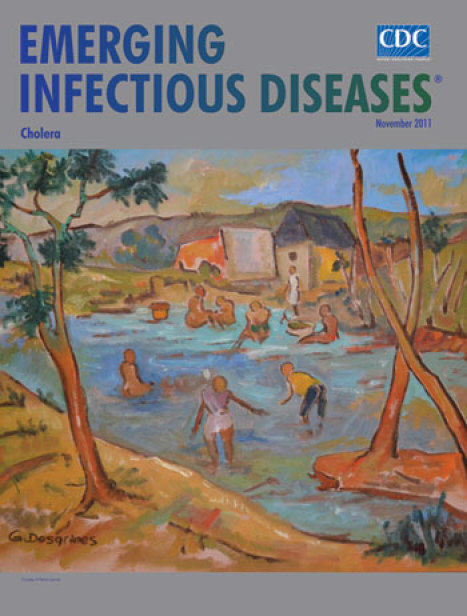
**Georges Desarmes (b. 1950) *The Bathers* (2006) (detail) Acrylic on canvas (61 cm × 50.8 cm)**. Courtesy of Patrick Lammie

“That anyone should condescend to die of cholera at the bidding of so insignificant a creature as the comma bacillus,” wrote Marcel Proust, should not be astonishing to those in the know. Plagued by illness from childhood, the author was very much in tune with medicine, which he pondered often in Remembrance of Things Past (1913), his monumental novel on the nature of memory. He also knew about cholera. His father, eminent physician and public health pioneer Achille-Andrien Proust, dedicated much of his life to promoting *cordon sanitaire* for the control of the disease, convinced that “questions of international hygiene reach beyond the borders established by politics.” The elder Proust’s idea, ahead of his time, challenged free trade so it was not until the Bombay cholera epidemic of 1877 that the concept of quarantine prevailed over commercialism and self-interest.

Marcel Proust was proud of his father. If visitors to the house were ever unwell, he was wont to ask them, “Would you like Papa to come to see you?” Once, when he made the offer to Anatole France, the man of letters replied, “My dear young friend, I should never dare to consult your father; I’m not important enough for him. The only patients he takes on nowadays are river basins!” Indeed, Dr. Proust had turned to public health. At great personal risk, he had cared for many a cholera patient during the 1866 epidemic in France and came to understand that individual treatment could not defeat this disease but prevention might control it. An early authority on epidemiology and a tireless advocate of an international sanitation system against disease spread, he is known in the history of medicine for his single-minded devotion to achieving the exclusion of cholera from the borders of Europe. This, some 20 years before Robert Koch identified the causative agent of the disease.

A severe infection spread by water contaminated with human waste, cholera was likely known in antiquity. Hippocrates and Galen described a compatible set of symptoms, and many sources point to similar illness, always present and frequently epidemic, in the plains of the Ganges River. In his landmark paper identifying the cause of the disease, Koch pinpointed the Ganges Delta as the *Heimat* (homeland) of cholera.

Many studied the scourge. In 1854, Filippo Pacini in Florence described vibrios in the intestinal contents of cholera victims and was amazed at their large numbers in the mucus and desquamated epithelial cells. He described the culprit as “an organic living substance of parasitic nature which can reproduce itself and thereby produce a specific disease.” In London, John Snow demonstrated the role of water as carrier of the disease, prompting local authorities to, reluctantly, remove the handle of the notorious Broad Street pump. But it was not until 1884, during the outbreak in Egypt, when Koch noted that the disease was a specific gastrointestinal infection caused by a comma-shaped bacillus, which he isolated in the laboratory and named *Vibrio cholerae*. And it was not until 1965 that the Judicial Committee on Bacteriological Nomenclature ruled that the organism should be known as *Vibrio cholerae* Pacini 1854.

Cholera can strike anywhere when sanitary conditions are compromised and the causative agent is present. After a short incubation period of 2 or 3 days, the patient becomes ill with serious diarrhea and nausea followed, in severe cases, by extreme dehydration and death. Early European observers were struck by the patients’ mummified appearance due to the draining of fluid from soft tissues.

Modern knowledge about cholera dates from the beginning of the 19th century, with seven major pandemics from 1800 to 1995, and has seen great progress in treatment if not prevention. With oral rehydration therapy, few patients should die if clean water is available. But floods and other natural disasters, along with social and economic ills favoring unsanitary conditions, compromise clean water supplies. Increased travel, population movements, and global conflict facilitate microbial traffic. Far from disappearing, cholera shows its ugly head as soon as the opportunity arises.

In October 2010, an outbreak of cholera was confirmed in Haiti. The two required conditions for emergence were present: *V. cholerae* introduced into the population and breaches in the water, sanitation, and hygiene infrastructure permitting exposure to contaminated water.

*The Bathers*, on this issue of Emerging Infectious Diseases, painted by Georges Desarmes, provides a glimpse of life on the Artibonite River and the bordering communities before the outbreak. Desarmes, born Yves Michaud in Port-au-Prince, began his artistic career working with Nehemy Jean, a well-traveled Haitian artist with diverse training in the United States and elsewhere. In the mid-1970s, Michaud met and went to work with Carlo Jean-Jacques, a Haitian impressionist; and in 2000, he started to paint in an entirely new style and assumed the name Georges Desarmes. Since then he has painted impressions of Haitian life.

In *The Bathers*, the artist captures the ease and communal charm of living along the river bank lined with tiny homes and populated with locals relating to each other on a personal level as they cool off. The scene is lyrical, spare, sunny, pre-cholera. On this day, this is the center of the universe, and this is the life. The artist captures the gist of it in a way only an impressionist could. As Proust would put it, “it’s a country to be happy in.” Yet this scene has since moved to another sphere, much like Proust’s impressionist moments in time.

Refusing to recognize false boundaries, cholera encompasses the frailties of political conflict and the aftermath of mass travel and increased human contact. Since no outbreaks were seen in the Caribbean since the mid-19th century, it was said that Haiti had no memory of or experience in handling cholera. But Proust would disagree. The memory was there: Ships pull in harbors with unknown pestilent cargo. Sanitary conditions are not optimal. Contraband microbes hop off and settle in new areas among populations with no immunity or infrastructure to prevent rapid spread of disease.

Desarmes’ lyrical impression of the waterfront meets Remembrance of Things Past. Proust struggled with the concept of involuntary memory, in which everyday cues evoke recollection of things past. But human history benefits more from voluntary memory, a deliberate effort to recall the past. Unless that happens, no one should be surprised if an inconsequential microbe causing a preventable and treatable disease continues to kill so many people.
